# Ubiquitin ligase UBE3C promotes melanoma progression by increasing epithelial-mesenchymal transition in melanoma cells

**DOI:** 10.18632/oncotarget.7393

**Published:** 2016-02-15

**Authors:** Li Tang, Xue-Mei Yi, Jia Chen, Fu-Juan Chen, Wei Lou, Yun-Lu Gao, Jing Zhou, Li-Na Su, Xin Xu, Jia-Qing Lu, Jun Ma, Ning Yu, Yang-Feng Ding

**Affiliations:** ^1^ Department of Dermatology, Shanghai Skin Disease Hospital, Shanghai, P. R. China; ^2^ Department of Pathology, Shanghai Skin Disease Hospital, Shanghai, P. R. China

**Keywords:** melanoma, invasion and metastasis, ubiquitin ligase, UBE3C

## Abstract

Melanoma is the most aggressive type of skin cancer, exhibiting extensive local invasion and early distant metastasis. Aberrant expression of ubiquitin-protein ligase E3C (UBE3C) plays a key role in tumor development and progression. In the present study, we analyzed UBE3C expression in samples of cancerous and normal skin tissue. Levels of UBE3C expression were much higher in primary and metastatic melanoma tissues than in normal skin, cutaneous squamous cell carcinoma or basal cell carcinoma. Melanoma cells overexpressing UBE3C frequently exhibited a mesenchymal phenotype, including reduced expression of the epithelial marker E-cadherin and expression of the mesenchymal marker vimentin. Knockdown of UBE3C expression in melanoma cells significantly suppressed melanoma growth and progression. Furthermore, silencing UBE3C led to increased E-cadherin expression and decreased vimentin and Snail1 expression. Thus UBE3C promotes melanoma progression, possibly by inducing epithelial-mesenchymal transition in melanoma cells. Inhibiting UBE3C activity may suppress melanoma invasion and metastasis and may represent a targeted therapeutic approach.

## INTRODUCTION

Melanoma is the most deadly form of skin cancer, and its incidence and mortality have been increasing in recent years [1, 2]. The long-term survival of melanoma patients remains poor [3], in large part reflecting an inability to stop the disease's progression and metastasis. Thus, understanding the molecular biology of melanoma progression could potentially facilitate the development of alternative therapeutic approaches based on blocking disease progression.

Numerous studies have shown that the ubiquitin proteasome system, especially the ubiquitin ligases, contribute to the progression of human malignancies, including melanoma [4–7]. For instance, ubiquitin-protein ligase E3C (UBE3C) is an important tumor-related regulatory molecule that promotes both tumor growth and metastasis [8–10]. In the present study, we investigated UBE3C expression in skin squamous cell cancer (SCC), skin basal cell cancer (BCC), primary melanoma (PM), and metastatic melanoma (MM). In addition, the functional relevance of UBE3C was further investigated using two human melanoma cell lines, A375 and SK-MEL-24. Associations between UBE3C and the epithelial-mesenchymal transition (EMT) markers E-cadherin and vimentin were also assessed in both melanoma tissues and cell lines. Our aim was to elucidate the association between UBE3C and melanoma progression and to identify an alternative therapeutic target for the treatment of melanoma.

## RESULTS

### Expression of UBE3C, E-cadherin and vimentin in skin cancer tissues

**Figure 1:**
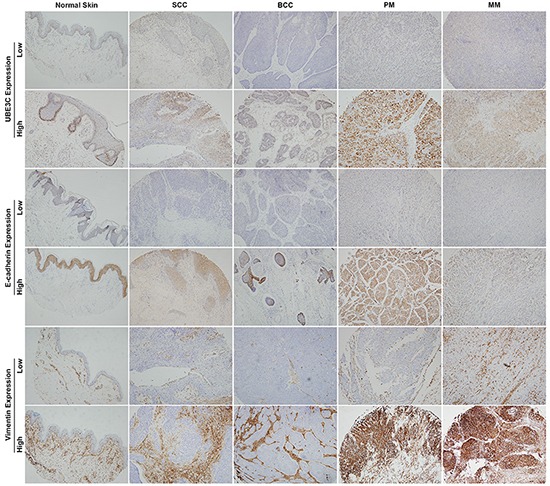
UBE3C, E-cadherin and vimentin expression in various skin cancer tissues and normal skin Primary and metastatic melanoma tissues showed higher UBE3C staining rates and stronger staining intensities than normal skin, SCC and BCC tissues.

To investigate the role of UBE3C in skin cancer, we first performed an immunohistochemical analysis of UBE3C using two skin cancer tissue microarrays containing samples of human skin cancer and normal skin tissues. Expression of UBE3C was detected mainly in the cytoplasm of melanoma cells. UBE3C was expressed in 9.5%, 7.1%, 73.2%, 62.5% and 10% of SCC, BCC, PM, MM and normal skin tissue samples, respectively (Table 1). Thus melanoma tissues, which have high metastatic capacity, showed the highest staining rate and the strongest intensity UBE3C staining. Normal skin and other skin cancers with low metastatic capacities (i.e., SCC and BCC) showed lower UBE3C staining levels, suggesting that UBE3C plays a role in melanoma metastasis (Figure 1).

**Table 1:** UBE3C, E-cadherin and vimentin expression in various skin cancer tissues and normal skin

Variable	UBE3C Expression, n (%)		E-cadherin Expression, n (%)		Vimentin Expression, n (%)	
	High	Low	High	Low	High	Low
**SCC (n=42)**	4 (9.5)	38 (90.5)	16 (38.1)	26 (61.9)	33 (78.6)	9 (21.4)
**BCC (n=14)**	1 (7.1)	13 (92.9)	2 (14.3)	12 (85.7)	12 (85.7)	2 (14.3)
**PM (n=41)**	30 (73.2)	11 (26.8)	20 (48.8)	21 (51.2)	34 (82.9)	7 (17.1)
**MM (n=16)**	10 (62.5)	6 (37.5)	5 (31.3)	11 (68.7)	11 (68.8)	5 (31.2)
**Normal Skin (n=10)**	1 (10.0)	9 (90.0)	9 (90.0)	1 (10.0)	5 (50.0)	5 (50.0)

Abbreviations: SCC, squamous cell cancer; BCC, basal cell cancer; PM, primary melanoma; MM, metastatic melanoma.

We also assessed the protein expression of the epithelial marker E-cadherin and the mesenchymal marker vimentin in the same human skin cancer tissue microarrays. Significantly lower levels of E-cadherin expression were found in 61.9%, 85.7%, 51.2%, 68.7% and 10% of SCC, BCC, PM, MM and normal skin tissue samples, respectively. Vimentin was expressed in 78.6%, 85.7%, 82.9%, 68.8% and 50% of SCC, BCC, PM, MM and normal skin tissues, respectively (Table 1 and Figure 1).

### Clinical relevance of UBE3C, E-cadherin and vimentin expression in skin cancer

We next examined the relationships between UBE3C, E-cadherin and vimentin expression and the clinicopathological features associated with skin cancer. UBE3C expression was significantly associated with SCC tumor grade (*P*=0.002), age of primary melanoma patients (*P*=0.011) and TNM stage (*P*=0.011). UBE3C expression was not associated with the gender of SCC or melanoma patients, patient age or TNM stage (Table 2). Levels of E-cadherin and vimentin expression did not correlate with the clinicopathological features of SCC or melanoma (Table 2).

**Table 2:** Clinical features of skin cancer patients and UBE3C, E-cadherin and vimentin expression

Variable	UBE3C Expression			E-cadherin Expression			Vimentin Expression		
	High	Low	P	High	Low	P	High	Low	P
**SCC**									
Gender									
Female	0	11	0.210	2	9	0.113	9	2	0.760
Male	4	27		14	17		24	7	
Age, years*	66.0±14.0	65.8±12.8	0.982	64.6±11.8	66.6±13.4	0.628	64.8±13.6	69.9±8.1	0.289
Tumor Grade									
1	1	23	0.002	12	12	0.128	19	5	0.586
2	1	14		4	11		11	4	
3	2	1		0	3		3	0	
TNM Stage									
I	0	11	0.576	7	4	0.084	8	3	0.215
II	4	25		8	21		24	5	
III	0	1		0	1		1	0	
IV	0	1		1	0		0	1	
**PM**									
Gender									
Female	11	2	0.260	6	7	0.819	11	2	0.845
Male	19	9		14	14		23	5	
Age, years*	49.8±10.8	61.6±16.7	0.011	56.2±16.7	49.9±9.0	0.135	53.6±13.7	49.9±13.3	0.513
TNM Stage									
I	0	3	0.011	3	0	0.090	2	1	0.602
II	26	6		14	18		26	6	
III	2	2		3	1		4	0	
IV	2	0		0	2		2	0	
**MM**									
Gender									
Female	6	3	0.696	4	5	0.197	6	3	0.838
Male	4	3		1	6		5	2	
Age, years*	51.0±13.2	49.0±8.2	0.745	47.6±11.5	51.5±11.6	0.546	53.3±12.5	43.6±3.4	0.116

* Mean±SD. Abbreviations: SCC, squamous cell cancer; PM, primary melanoma; MM, metastatic melanoma.

### Correlations between UBE3C and EMT markers in melanoma tissues

**Figure 2:**
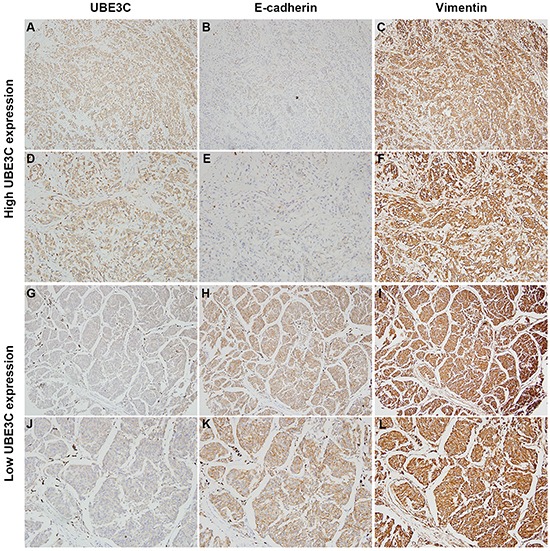
Correlation between UBE3C, E-cadherin and vimentin expression in melanoma tissues Immunohistochemical analyses indicated that melanoma tissues with high UBE3C expression frequently also had low E-cadherin and high vimentin expression in the same patients. Shown are representative cases with UBE3C^high^/E-cadherin^low^/vimentin^high^ (**A, B, C**, 100×; **D, E, F**, 200×) and UBE3C^low^/E-cadherin^high^/vimentin^low^ (**G, H, I**, 100×; **J, K, L**, 200×) expression profiles.

We also observed that melanoma cells overexpressing UBE3C exhibited a mesenchymal phenotype. Moreover, a higher proportion of melanoma tissues exhibited a UBE3C^high^/E-cadherin^low^/vimentin^high^ expression profile in which high levels of UBE3C expression were frequently accompanied by low levels of E-cadherin expression (Figure 2). Statistical analysis revealed significant inverse correlations between UBE3C and E-cadherin expression in both PM and MM tissues (Table 3).

**Table 3:** Correlations between UBE3C and E-cadherin expression in primary and metastatic melanoma tissues

Variable	UBE3C Expression		
	High	Low	P
**PM (n=41)**			
**E-cadherin Expression**			
**Low**	19	2	0.010
**High**	11	9	
**MM (n=16)**			
**E-cadherin Expression**			
**Low**	9	2	0.018
**High**	1	4	

Abbreviations: PM, primary melanoma; MM, metastatic melanoma.

### Function of UBE3C in melanoma cells

**Figure 3:**
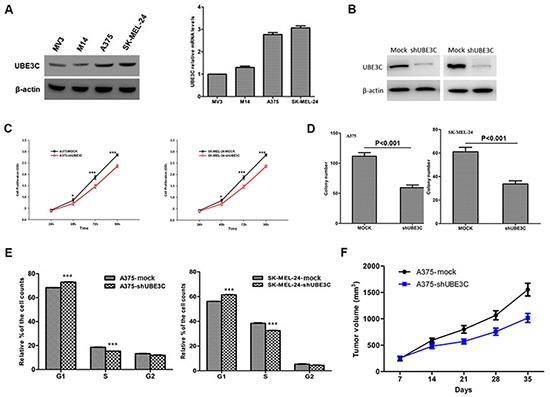
Functional analyses of UBE3C in A375 and SK-MEL-24 cells **A.** Expression of UBE3C protein and mRNA in the indicated melanoma cell lines. **B.** Western blots showing the efficacy of UBE3C knockdown. **C.** UBE3C knockdown significantly decreased melanoma cell growth in CCK-8 assays. **D.** UBE3C knockdown significantly inhibited melanoma cell proliferation in colony formation assays. **E.** Flow cytometric analysis of the cell cycle distribution among melanoma cells after UBE3C knockdown. **F.** UBE3C knockdown suppressed melanoma growth in a subcutaneous xenograft tumor model. *P < 0.05; ***P < 0.001.

**Figure 4:**
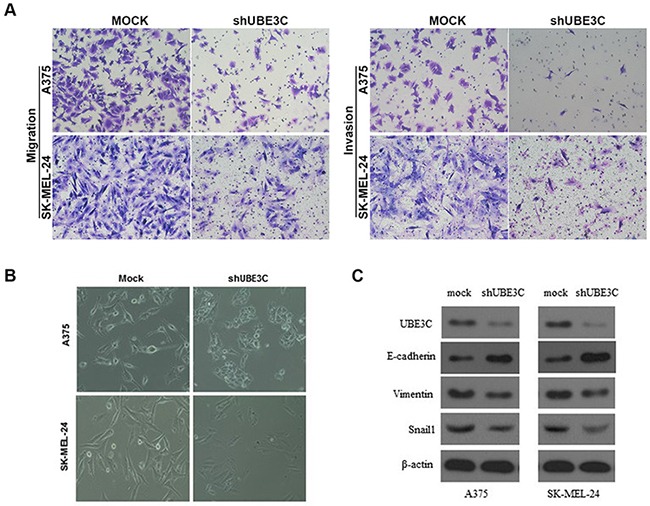
Relationship between UBE3C and melanoma progression **A.** UBE3C knockdown significantly inhibited melanoma cell migration and invasion in Matrigel transwell assays. Permeating cells were stained with Giemsa and counted. **B.** Morphological differences between A375-mock and SK-MEL-24-mock cells and UBE3C-knockdown cells. **C.** UBE3C knockdown led to up-regulation of E-cadherin and down-regulation of vimentin and Snail1 in melanoma cells.

To investigate the function of UBE3C in melanoma, we used the A375 and SK-MEL-24 human melanoma cell lines to assess the impact of RNA interference (RNAi)-mediated UBE3C knockdown on the behavior melanoma cells. We found that UBE3C knockdown in both cell lines significantly suppressed cell proliferation, migration and invasion in vitro and tumor growth in vivo (Figure 3 and 4A). We also found that UBE3C knockdown resulted in the up-regulation of E-cadherin and down-regulation of vimentin and Snail1 (Figure 4B, 4C). These results suggest that inhibiting UBE3C expression may inhibit EMT in melanoma cells.

## DISCUSSION

Skin cancer is one of the most common human cancers, and melanoma is the most aggressive type, exhibiting locoregional invasiveness and early distant dissemination [11]. The need to understand the molecular biology of melanoma invasion and metastasis has fueled intensive investigation. Ubiquitin ligases critically influence melanoma progression by binding highly selective substrate proteins required for ubiquitin-mediated proteolysis, which is essential for maintaining cellular homeostasis [5–7]. The aberrant expression of ubiquitin ligases can lead to a malignant cellular phenotype and even accelerate cancer metastasis [12, 13]. The association between the ubiquitin ligase UBE3C and tumor progression was recently established by several studies [8–10]. UBE3C is reportedly involved in the growth and metastasis in several types of solid tumors, possibly by inducing EMT, activating the Wnt/β-catenin pathway, and/or degrading various tumor-related proteins [14–16].

In the present study, we analyzed the expression of UBE3C and two typical EMT markers, E-cadherin and vimentin, in skin cancer and normal skin tissues. UBE3C levels were higher in both primary and metastatic melanoma than in normal skin and other skin cancers (SCC and BCC). Moreover, we observed a link between UBE3C overexpression and reduced E-cadherin expression in both primary and metastatic melanoma. Subsequent functional experiments showed that UBE3C promoted melanoma progression by inducing EMT. Inhibiting UBE3C expression increased E-cadherin expression. These results suggest that UBE3C promotes melanoma progression by inducing EMT, perhaps by decreasing E-cadherin expression.

EMT is considered a crucial step during the metastatic spread of cancer cells. Reduction of E-cadherin expression is a fundamental event in EMT and plays an important part in producing single migratory cells descended from cancerous epithelial-like cells [17–20]. Our results are consistent with numerous studies showing significant reductions in E-cadherin expression in melanoma tissues [21]. Furthermore, we observed a significant inverse correlation between UBE3C and E-cadherin expression. Melanoma tissues with high UBE3C expression frequently showed low E-cadherin expression in the same patients. Melanoma cells that overexpressed UBE3C exhibited a mesenchymal phenotype, and UBE3C reduced the expression of E-cadherin in melanoma cells. These findings imply that UBE3C may act as an EMT inducer that accelerates melanoma development and progression, possibly by targeting E-cadherin for ubiquitin-mediated degradation. Thus inhibiting UBE3C expression may not only inhibit the growth and migration of melanoma cells, it may also restore E-cadherin expression, reverse the EMT phenotype, and attenuate the metastasis of melanoma cells. However, further study will be required to clarify the specific relationship between UBE3C and E-cadherin, which functionally regulates the EMT process in melanoma cells.

In summary, the ubiquitin ligase UBE3C is overexpressed in both primary and metastatic melanoma tissues, where it plays a key role in disease development and progression. UBE3C may be an alternative target for melanoma therapy.

## MATERIALS AND METHODS

### Skin cancer tissue microarrays

Human skin squamous cell carcinoma tissue (SK801b) and malignant melanoma tissue (ME483a) microarrays were purchased from US Biomax Inc. (Rockville, MD, USA). The microarrays contained 42, 14, 41, 16, and 10 cases of SCC, BCC, PM, MM, and normal skin (a single core per case), respectively. The PM cases included 37 skin melanomas, 2 soft tissue melanomas, 1 fibrous tissue melanoma, and 1 adipose tissue melanoma. The MM cases included 16 lymph node metastases. Each array spot was 1.5 mm in diameter and 5 μm thick. Detailed information pertaining to these microarrays can be viewed at http://www.biomax.us/tissue-arrays/Skin/SK801b and http://www.biomax.us/tissue-arrays/Melanoma/ME483a.

### Cell lines and plasmids constructs

The A375, SK-MEL-24, MV3 and M14 human melanoma cell lines were obtained from the Cell Bank of the Shanghai Institutes of Biological Sciences, Chinese Academy of Sciences. Plasmid constructs were created and cells were transfected as described previously [8].

### Cell proliferation, migration, matrigel invasion, flow cytometry and in vivo assays

The cell counting kit-8 (CCK-8), colony formation, Matrigel Transwell, flow cytometry and in vivo assays were all performed as described previously [22, 23].

### Western blotting and immunohistochemical assays

Western blotting and immunohistochemistry procedures were performed as described previously [8]. For immunohistochemical analysis, tissue microarrays were stained with specific primary antibodies against UBE3C (HPA039915, Sigma Aldrich), E-cadherin, vimentin and Snail1 (Cell Signaling Technology, Beverly, MA). Staining was independently assessed by two experienced pathologists. Staining intensities were graded as described previously [8].

### Statistical analysis

Statistical analyses were performed using SPSS 19.0 software (SPSS, IBM). Student's *t* test and the χ2 test were used as appropriate. Values of *P* < 0.05 were considered statistically significant.

## Abbreviations

SCC: squamous cell cancer
BCC: basal cell cancer
PM: primary melanoma
MM: metastatic melanoma
EMT: epithelial-mesenchymal transition
